# Adjuvant chemotherapy is associated with an overall survival benefit regardless of age in ER+/HER2- breast cancer pts with 1-3 positive nodes and oncotype DX recurrence score 20 to 25: an NCDB analysis

**DOI:** 10.3389/fonc.2023.1115208

**Published:** 2023-04-24

**Authors:** Nickolas Stabellini, Lifen Cao, Christopher W. Towe, Xun Luo, Amanda L. Amin, Alberto J. Montero

**Affiliations:** ^1^ Case Western Reserve University School of Medicine, Case Western Reserve University, Cleveland, OH, United States; ^2^ Faculdade Israelita de Ciências da Saúde Albert Einstein, Hospital Israelita Albert Einstein, São Paulo, Brazil; ^3^ Division of Hematology and Oncology, Department of Medicine, University Hospitals Seidman Cancer Center, University Hospitals Cleveland Medical Center, Cleveland, OH, United States; ^4^ Division of Thoracic and Esophageal Surgery, Department of Surgery, University Hospitals Cleveland Medical Center, Cleveland, OH, United States; ^5^ University Hospitals Research in Surgical Outcomes and Effectiveness (UH-RISES), University Hospitals Cleveland Medical Center, Cleveland, OH, United States; ^6^ Division of Surgical Oncology, Department of Surgery, University Hospitals Seidman Cancer Center, University Hospitals Cleveland Medical Center, Cleveland, OH, United States

**Keywords:** adjuvant (chemo)radiotherapy, chemotherapy, breast cancer, ER+ breast cancer, HER2- breast cancer, survival, oncotype

## Abstract

**Background:**

The RxPONDER trial found that among breast cancer patients with estrogen receptor positive (ER+) breast cancer, 1-3 positive axillary nodes, and a recurrence score of ≤25, only pre-menopausal women benefitted from adjuvant chemoendocrine therapy; postmenopausal women with similar characteristic did not benefit from adjuvant chemotherapy. We aimed to replicate the RxPonder trial using a larger patient cohort with real world data to determine whether a RS threshold existed where adjuvant chemotherapy was beneficial regardless of age.

**Methods:**

The National Cancer Database (NCDB) was queried for women with ER+, human epidermal growth factor receptor 2 (HER2) negative breast cancer, 1-3 positive axillary nodes, and RS ≤25 who received endocrine (ET) only or chemo-endocrine therapy (CET). Cox regression interaction was explored between CET and age as a surrogate for menopausal status.

**Results:**

The final analytic cohort included 28,427 eligible women: 7,487 (26.3%) received adjuvant CET and 20,940 (73.7%) ET. In the entire cohort, RS had a normal distribution, with a median score of 14. After correcting for demographic and clinical variables, a threshold effect was observed with RS >20 being associated with a significantly inferior overall survival (OS) (P value range: < 0.001-0.019). In women with RS of 20-25, CET was associated with a significant improvement in OS compared to ET alone, regardless of age (age <=50: HR = 0.334, P=0.002; age>50: HR=0.521, P=0.019).

**Conclusion:**

Among women with ER+/HER2- breast cancer with 1–3 positive nodes, and a RS of 20-25—in contrast to the RxPONDER trial—we observed that CET was associated with an OS benefit in women regardless of age.

## 1 Introduction

The 21-gene assay Oncotype DX^®^ Breast Recurrence Score (RS), has been used widely to guide adjuvant chemotherapy utilizations in patients with estrogen receptor (ER)+/human epidermal growth factor receptor 2 (HER2) negative node-negative breast cancer (BC) (1–4), and are part of international consensus guidelines. In patients with axillary nodal metastasis, RS has also been demonstrated to identify which patients can safely forgo adjuvant chemotherapy when they are post-menopausal. Evaluation from RxPONDER (SWOG S1007) trial comparing endocrine therapy alone (ET) *vs*. chemotherapy in addition to endocrine therapy (CET) in patients with 1–3 positive axillary lymph nodes and RS ≤ 25 found that CET did not improve distant recurrence free survival compared to ET in postmenopausal women with RS 0-25, regardless of clinical features. By contrast, CET was found to be beneficial in premenopausal women in this trial regardless of RS (5).

Our aim in this study was to replicate the RxPONDER trial using real world data and larger sample size from National Cancer Database (NCDB) to determine whether a RS threshold could be identified where CET was beneficial regardless of age.

## 2 Methods

### 2.1 Data collection and data elements

A retrospective cohort study of the NCDB was performed. Jointly sponsored by the American College of Surgeons and the American Society, NCDB is a clinical oncology database sourced from hospital registry data representing more than 70% of newly diagnosed BC cases nationwide. The database covers more than 1,500 Commission on Cancer (CoC)-accredited facilities. Definition of the database variables are available from the dictionary of NCDB Participant Use Data File (http://ncdbpuf.facs.org). The CoC’s NCDB and the hospitals participating in the CoC NCDB are the source of the de-identified data used herein; they have not verified and are not responsible for the statistical validity of the data analysis or the conclusions derived by the authors.

### 2.2 Patient cohort and data analysis

The NCDB was queried to identify HR+HER2- BC patients who underwent definitive breast surgery and had 1 to 3 positive axillary lymph nodes from 2004-2018. Clinical staging data for the cohort was based on TNM classification in American Joint Committee on Cancer (AJCC) 7th edition. Patients were excluded if they were stage 0 or stage IV, male, had RS > 25, or if they were missing critical study information (e.g. follow-up data or RS).

The cohort was divided by patients who received CET and ET. The primary outcome was overall survival (OS). Analysis included univariate comparison of patient factors associated with receipt of CET (*vs*. ET). Logistic regression analysis was performed to identify clinical factors that were predictive of CET. To compare the two groups, Wilcoxon rank-sum test was utilized for continuous variables and chi-square for categorical data. Difference in OS between the groups was analyzed using Kaplan-Meier survival estimates and compared through log-rank test. To control for confounding effects, multivariable Cox proportional hazard analysis was performed. The covariates included were: age, gender, race, insurance provider, facility, and patient clinical characteristics. Interaction between menopausal status (age <50 or above) and CET receipt was explored.

All statistical analysis was performed using STATA/MP, version 16.0 (Stata Corp LLC, College Station, TX). Institutional Review Board (IRB) approval was exempted by the University Hospitals Cleveland Medical Center IRB as all NCDB data is de-identified and does not contain any protected health information.

## 3 Results

The final analytic cohort included 28,427 women with a primary diagnosis of pathological stage I-III HR+HER2- BC with 1–3 positive axillary lymph nodes, i.e. pN1 (**Table 1**). The median follow-up time was 52.7 months (interquartile range [IQR] 35.3-74.3 months), 7,487 patients (26.3%) received CET and 20,940 (73.7%) received ET. Patients who received ET were more likely to be older (median age 61 *vs*. 54, P=0.001), White (87.1% *vs*. 86.5%, P=0.045), have non-private insurance (44.5% *vs*. 26.5%, P<0.001), have a greater number of comorbidities (1+ Charlson-Deyo Score 16.6% *vs*. 12.6% P<0.001), and a lower RS (<11, 37.9% *vs* 17.0%, P<0.001) compared to patients who received CET.

**Table 1:** Demographic, clinical and treatment characteristics of pathological stage I-III HR+HER2- breast cancer patients with 1 – 3 positive nodes, NCDB 2004-2018.

CHARACTERISTICS	Endocrine therapy alone (n=20,940)		Endocrine therapy plus chemotherapy (n= 7,487)		
	NO.	%	NO.	%	*P* value
Age, years	61 (20-90)		54(32-84)		P<0.001
Age≤50	3,954	18.88	2,903	38.77	P<0.001
Age>50	16,986	81.12	4,584	61.23	
Oncotype					P<0.001
≤11	7,941	37.92	1,271	16.98	
12-25	12,999	62.08	6,216	83.02	
Race					P=0.045
White	18,074	87.11	6,426	86.5	
Black	1,679	8.09	592	7.97	
Asian and other	996	4.8	411	5.53	
Charlson-Deyo Score					<0.001
0	17,468	83.42	6,546	87.43	
1	2,753	13.15	779	10.4	
2	525	2.51	130	1.74	
3	194	0.93	32	0.43	
Insurance					P<0.001
Public	8,951	43.14	1,859	25.09	
Private	11,515	55.5	5,444	73.47	
Not insured	282	1.36	107	1.44	
Facility Type					P<0.001
Community cancer program	1,194	5.8	388	5.55	
Comprehensive community cancer program	8,375	40.66	2,608	37.33	
Academic/research program	6,480	31.46	2,488	35.61	
Integrated network cancer program	4,548	22.08	1,503	21.51	
Facility Area					P=0.022
Metro	17,638	86.42	6,342	87.66	
Urban	2,503	12.26	799	11.04	
Rural	269	1.32	94	1.3	
Grade					P<0.001
Well differentiated	6,382	31.46	1,574	21.82	
Moderately differentiated	12,193	60.1	4,516	62.61	
Poorly differentiated	1,703	8.39	1,120	15.53	
Undifferentiated	10	0.05	3	0.04	
Lymphovascular invasion					P<0.001
Not Present	12,638	69.22	3,954	60.47	
Present	5,620	30.78	2,585	39.53	
Positive Nodes					P<0.001
1	16,956	80.97	5,188	69.29	
2	3,200	15.28	1,666	22.25	
3	784	3.74	633	8.45	
Pathological stage					P<0.001
I	5,366	25.99	1,108	15.05	
II	14,722	71.31	5,859	79.56	
III	557	2.7	397	5.39	
Breast Surgery Type					P<0.001
Partial mastectomy	13,228	63.17	4,040	53.96	
Unilateral mastectomy	5,208	24.87	1,998	26.69	
Bilateral mastectomy	2,504	11.96	1,449	19.35	
Axillary Surgery Type					P<0.001
SLNB (1-5 lymph nodes)	13,410	64.14	3,895	52.08	
ALND (>5 lymph nodes)	7,499	35.86	3,584	47.92	

Multivariable logistic regression was performed to determine patient and clinical characteristics that were independently associated with CET *vs*. ET (**Table 2**). After accounting for available demographic and clinical-pathological factors, patients with the following factors were more likely to receive CET: higher RS (OR = 2.8, 95% confidence interval [CI] 2.6-3.0, P<0.001), grade 2-3 BC (OR = 1.5, 95% CI 1.3-1.6, P<0.001), lympho-vascular invasion (OR = 1.2, 95% CI 1.1-1.3, P<0.001), and private insurance (OR = 1.2, 95% CI 1.1-1.3, P<0.001). Conversely, age was inversely related to likelihood of receipt of CET (OR = 0.9, 95% CI 0.9-0.9, P<0.001).

**Table 2:** Multivariable logistic regressions for predictors of receipt of chemotherapy in pathological stage I-III HR+HER2- breast cancer patients with 1 – 3 positive nodes, NCDB 2004-2018.

	Odds Ratio	95% Conf. Int.		p-value
Age	0.944	0.940	0.948	<0.001
RS 12-25 *vs*. 0-11	2.829	2.613	3.064	<0.001
Race				
White	Reference			
African American	0.910	0.806	1.028	0.130
Asian or others	0.984	0.852	1.138	0.832
Charlson-Deyo score				
0	Reference			
1	0.958	0.865	1.061	0.411
2	0.903	0.716	1.139	0.389
3	0.674	0.439	1.034	0.071
Facility Type				
Community	Reference			
Comprehensive	0.857	0.740	0.992	0.039
Academic	0.964	0.831	1.118	0.627
Integrated	0.877	0.752	1.023	0.094
Insurance Status				
Public insurance	Reference			
Private insurance	1.176	1.082	1.278	<0.001
Not insured	0.872	0.653	1.163	0.351
Grade				
Well differentiated	Reference			
Moderately differentiated	1.471	1.361	1.590	<0.001
Poorly or undifferentiated	2.438	2.176	2.731	<0.001
Lympho-vascular invasion	1.238	1.156	1.325	<0.001
Pathological stage				
I	Reference			
II	2.154	1.977	2.346	<0.001
III	4.178	3.503	4.983	<0.001

**Figure 1:**
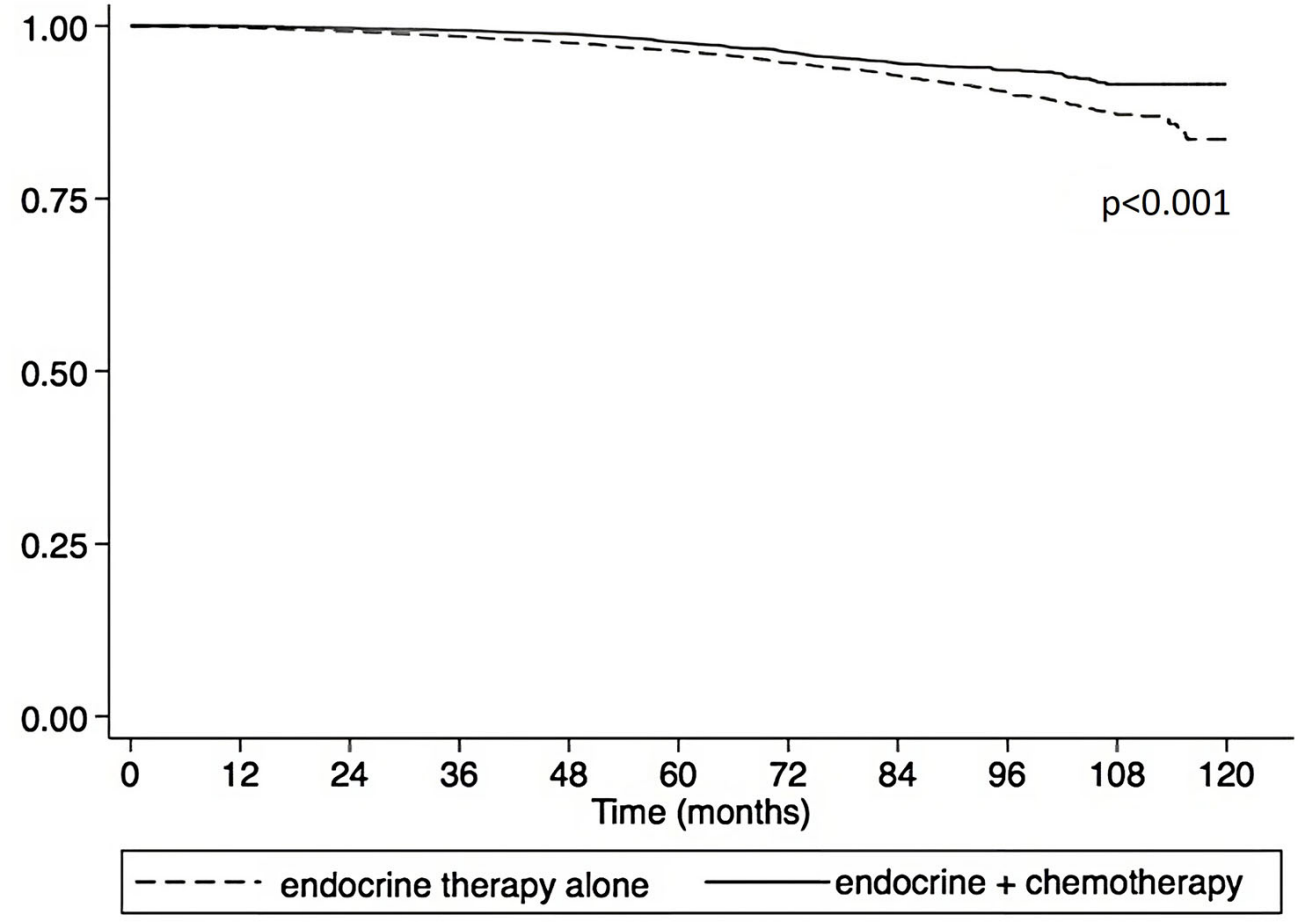
Overall survival compared between endocrine therapy alone versus endocrine therapy plus chemotherapy in pathological stage I-III HR+HER2- breast cancer patients with 1 – 3 positive nodes and RS 0-25. (OS at 3 and 5 years were 98.5% and 96.4% for the endocrine therapy alone cohort compared to 99.3% and 97.6%, respectively for the endocrine plus chemotherapy group (P<0.001).

**Figure 2:**
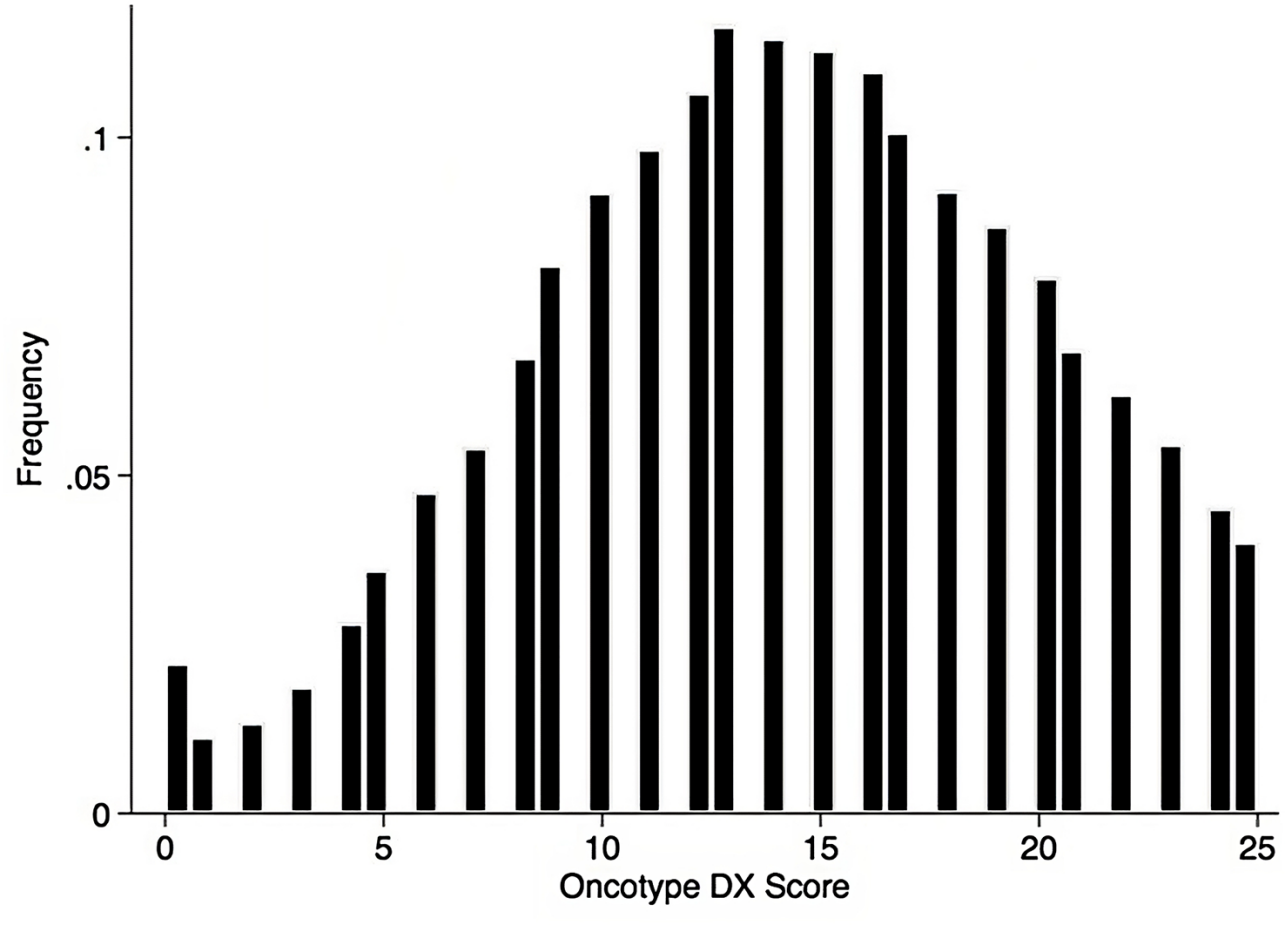
Histogram of RS among the pathological stage I-III HR+HER2- breast cancer patients with 1 – 3 positive nodes, data shown as percent of patients.

**Figure 3:**
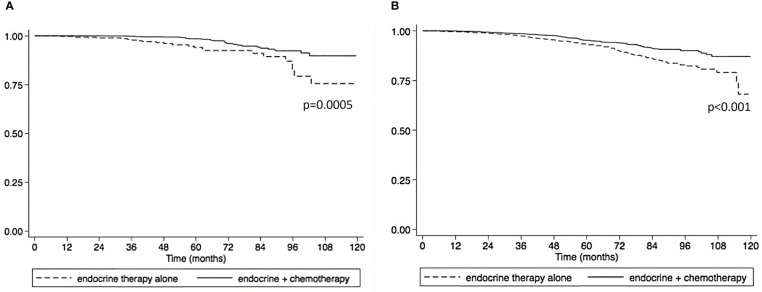
Overall survival compared between endocrine therapy alone versus endocrine therapy plus chemotherapy in pathological stage I-III HR+HER2- breast cancer patients with 1 – 3 positive nodes and RS 20-25. **(A)** premenopausal patients. **(B)** postmenopausal patients).

Using the Kaplan-Meier estimate, OS was superior in CET compared to ET in the entire cohort (P<0.001, **Figure 1**). In the entire cohort, RS had a normal distribution (**Figure 2**), with a median RS of 14. To further explore the relationship of RS and OS benefit with receipt of CET, a multivariate Cox regression was performed with each individual RS of 11-25 (**Table 3**). After correcting for demographic and clinical features, we observed a threshold effect as patients with RS of >20 had a significantly inferior OS (P value ranged from <0.001-0.019). Patients were divided into two groups using a RS of 19 as a cut-off, and examined whether any interactions existed between CET and age as a surrogate for menopausal status. Among patients with RS of 0-19, CET was not associated with a significantly improved OS when compared to ET (**Table 4**) regardless of age (≤50, P=0.068; >50, P=0.770). By contrast, in women with RS of 20-25, the combination CET was associated with a significant improvement in OS compared to ET alone, regardless of age (HR = 0.334, P=0.002 for age ≤50, and HR=0.521, P=0.019 for age >50, **Table 4** and **Figure 3**). In the subgroup of women over 50 and a RS of 20-25, CET was associated with a significant improvement in OS (HR = 0.84, 95% CI 0.5-0.9, p=0.038) compared to ET (**Supplemental Table 1**).

**Table 3:** Cox proportional hazard regression for overall survival pathological stage I-III HR+HER2- breast cancer patients with 1 – 3 positive nodes and individual RS 11-25.

	Hazards Ratio	95% Conf. Int.		p-value
Chemotherapy *vs*. endocrine therapy alone	0.659	0.542	0.799	<0.001
Age ≤50 *vs*. Age >50	1.421	1.093	1.846	0.009
Race				
White	Reference			
African American	1.128	0.877	1.451	0.350
Asian or others	0.518	0.299	0.900	0.020
Charlson-Deyo score				
0	Reference			
1	1.673	1.375	2.035	<0.001
2	3.287	2.427	4.452	<0.001
3	5.265	3.539	7.835	<0.001
Facility Type				
Community	Reference			
Comprehensive	0.836	0.617	1.133	0.249
Academic	0.621	0.452	0.855	0.003
Integrated	0.587	0.420	0.820	0.002
Insurance Status				
Public insurance	Reference			
Private insurance	0.426	0.358	0.507	<0.001
Not insured	0.896	0.490	1.640	0.723
Grade				
Well differentiated	Reference			
Moderately differentiated	1.200	0.986	\]1.461	0.069
Poorly or undifferentiated	1.660	1.290	2.137	<0.001
Lympho-vascular invasion	1.137	0.965	1.338	0.125
Pathological stage				
I	Reference			
II	2.125	1.668	2.707	<0.001
III	2.970	1.940	4.546	<0.001
Oncotype DX score				
11	Reference			
12	1.126	0.731	1.735	0.590
13	1.033	0.670	1.592	0.884
14	1.338	0.890	2.010	0.162
15	0.963	0.617	1.504	0.869
16	1.153	0.748	1.778	0.519
17	1.450	0.958	2.195	0.079
18	0.917	0.571	1.473	0.722
19	1.523	0.984	2.357	0.059
20	1.705	1.107	2.626	0.015
21	2.198	1.455	3.319	<0.001
22	1.965	1.269	3.042	0.002
23	1.792	1.100	2.921	0.019
24	2.021	1.274	3.206	0.003
25	2.530	1.581	4.050	<0.001

Table 4ACox proportional hazard regression for overall survival pathological stage I-III HR+HER2- breast cancer patients with 1 – 3 positive nodes and RS 0-19.

**Table:** 

	Hazards Ratio	95% Conf. Int.		p-value
Race				
White	Reference			
African American	1.219	0.935	1.589	0.143
Asian or others	0.354	0.176	0.712	0.004
Charlson-Deyo score				
0	Reference			
1	1.848	1.509	2.264	<0.001
2	3.611	2.656	4.908	<0.001
3	5.395	3.628	8.022	<0.001
Facility Type				
Community	Reference			
Comprehensive	1.027	0.725	1.455	0.882
Academic	0.760	0.528	1.094	0.140
Integrated	0.784	0.539	1.139	0.201
Insurance Status				
Public insurance	Reference			
Private insurance	0.391	0.324	0.471	<0.001
Not insured	0.805	0.398	1.627	0.546
Grade				
Well differentiated	Reference			
Moderately differentiated	1.203	0.992	1.459	0.061
Poorly or undifferentiated	1.555	1.171	2.065	0.002
Lympho-vascular invasion	1.085	0.913	1.291	0.354
Pathological stage				
I	Reference			
II	2.049	1.606	2.614	<0.001
III	2.788	1.781	4.364	<0.001
Age and treatment interactions				
Age ¾50 and endocrine tderapy alone	Reference			
Age ¾50 and endocrine plus chemotherapy	0.560	0.301	1.043	0.068
Age > 50 and endocrine therapy alone	1.605	1.126	2.287	0.009
Age>50 and endocrine plus chemotherapy	1.065	0.697	1.628	0.770

**Table 4B:** Cox proportional hazard regression for overall survival pathological stage I-III HR+HER2- breast cancer patients with 1 – 3 positive nodes and RS 20-25.

	Hazards Ratio	95% Conf. Int.		p-value
Race				
White	Reference			
African American	1.034	0.687	1.555	0.873
Asian or others	0.705	0.330	1.504	0.366
Charlson-Deyo score				
0	Reference			
1	1.475	1.070	2.032	0.018
2	2.594	1.516	4.439	0.001
3	2.840	1.155	6.983	0.023
Facility Type				
Community	Reference			
Comprehensive	0.638	0.405	1.004	0.052
Academic	0.502	0.312	0.809	0.005
Integrated	0.464	0.279	0.770	0.003
Insurance Status				
Public insurance	Reference			
Private insurance	0.444	0.335	0.587	<0.001
Not insured	0.925	0.339	2.524	0.879
Grade				
Well differentiated	Reference			
Moderately differentiated	1.179	0.825	1.684	0.367
Poorly or undifferentiated	1.902	1.266	2.858	0.002
Lympho-vascular invasion	1.096	0.842	1.427	0.496
Pathological stage				
I	Reference			
II	2.533	1.669	3.844	<0.001
III	2.457	1.058	5.704	0.036
Age and treatment interactions				
Age ¾50 and endocrine therapy alone	Reference			
Age ¾50 and endocrine plus chemotherapy	0.334	0.166	0.670	0.002
Age > 50 and endocrine therapy alone	0.712	0.415	1.220	0.216
Age>50 and endocrine plus chemotherapy	0.521	0.302	0.898	0.019

Using a RS of 11 as a cutoff to examine the interaction between CET and menopausal status, we found that CET was associated with a significant improvement in OS —using age as a surrogate for premenopausal status — in women 50 and under with an RS of 12-25 (**Supplemental Tables 2A, B**).

## 4 Discussion

The Early Breast Cancer Trialists’ Collaborative Group (EBCTCG) have consistently shown in multiple meta-analyses that adjuvant chemotherapy significantly reduces cancer related mortality—a benefit that is independent of age (6, 7). In the 2005 meta-analysis the EBCTCG reported that in women with ER+ BC anthracycline-based adjuvant poly-chemotherapy reduced annual BC death rates by 38% in women younger than 50 years of age at time of initial diagnosis, and by about 20% for women 50–69 years when diagnosed. This benefit of adjuvant chemotherapy was observed largely irrespective of the use of tamoxifen and of ER status, nodal status, or other tumor characteristics.

This differential benefit of adjuvant chemotherapy in younger pre-menopausal *vs*. older post-menopausal women is possibly related to an indirect anti-estrogen effect, by the ability of chemotherapy to induce premature ovarian failure. The Zebra trial demonstrated in premenopausal women with ER-positive and node-positive early stage BC, that ovarian ablation with goserelin provided a benefit which was similar to that of adjuvant CMF chemotherapy (8).

Therefore, it was somewhat unexpected that in RxPONDER in post-menopausal women with pN1 disease no significant benefit in OS of CET was observed. Our study, by contrast was more consistent with results of EBCTCG, in that among women >50 with ER+/HER2- BC with 1–3 positive nodes, and a RS of 20-25, CET was associated with an OS benefit. These results using a real world cohort of patients from NCDB suggests that women >50, many of whom are presumably post-menopausal, with a RS of 20-25 appear to still derive an OS benefit from CET compared to patients who received ET.

Similarly Pagani et al. demonstrated that CET was associated with a significantly improved disease-free survival among postmenopausal women with ER-positive, node-positive breast cancer— although the magnitude of the benefit was less in patients highly ER+ tumors, with only 1 axillary lymph node, or in older women (9). Similarly, in a retrospective analysis of SWOG-8814 a significant benefit from adjuvant anthracycline based CET was reported by Albain et al. (10) in postmenopausal women with node-positive, ER+ BC, and RS >31. Interestingly, in the 103 women with intermediate RS (18–30), although the number of events was small, there was a trend towards an improved DFS with CET *vs*. ET (HR=0.72; 95% CI 0.39−1.31) which improved over time. In our study, we did observe a threshold effect with RS of 20 and above as associated with an inferior OS which was statistically significant (P value ranged from < 0.001 to 0.019).

Our study does have numerous limitations, despite the advantages of the large sample size and long follow-up times. Firstly, because the NCDB does not include local regional recurrence and disease-free survival, one major limitation of our study is that our analysis of long-term outcomes was limited to OS. Another limitation in our study is that we used age >50 as a surrogate for menopause status since NCDB does not specifically define menopausal status. However, age is not a therapeutic target and many women in their fifties maintain ovarian function. Therefore, a persistent endocrine effect of cytotoxics of chemotherapy may produce a larger impact of chemotherapy in younger postmenopausal women (less than 60 years) (9). Similarly, we do not have access to detailed granular data on the precise steroid hormone receptor concentrations (ER or PR) in the primary tumor or specific chemotherapeutic or ET treatment regimens which therefore cannot be factored into analyses. Finally, the NCDB only receives data from Commission on Cancer (CoC) accredited hospitals, and therefore excludes patients treated in many non-CoC accredited centers in the United States. Despite these limitations, these data suggest that there is a sub-population of postmenopausal women with RS 20-25 who appear to benefit from CET.

In summary, among women with ER+/HER2- BC with 1–3 positive axillary lymph nodes, and a RS of 20-25—in contrast to the RxPONDER—we observed that CET was associated with an OS benefit in women regardless of age underscoring that there could be hormone independent anti-tumor effects of chemotherapy in ER+ breast cancer.

## Data availability statement

Publicly available datasets were analyzed in this study. This data can be found here: https://www.facs.org/quality-programs/cancer-programs/national-cancer-database/.

## Author contributions

LC and AM contributed to the conception or design of the work. LC, NS, XL and CT contributed to the acquisition, analysis, or interpretation of data for the work. LC drafted the work. LC, NS, AA, AM and CT revised it critically for important intellectual content. LC, NS, CT, XL, AA and AM approved the final version to be published and agreed to be accountable for all aspects of the work in ensuring that questions related to the accuracy or integrity of any part of the work are appropriately investigated and resolved. All authors contributed to the article and approved the submitted version.
