# Supplementary material for: Adjuvant chemotherapy is associated with an overall survival benefit regardless of age in ER+/HER2- breast cancer pts with 1-3 positive nodes and oncotype DX recurrence score 20 to 25: an NCDB analysis
Source: Front Oncol. 2023 Apr 24;13:1115208. doi: 10.3389/fonc.2023.1115208 (PMC10165881; doi:10.3389/fonc.2023.1115208)
Supplement: Supplementary file 1 [file Table_1.docx]

Supplementary Material

Adjuvant Chemotherapy is associated with an overall survival benefit regardless of age in ER+/HER2- breast cancer pts with 1-3 positive nodes and Oncotype DX recurrence score 20 to 25: An NCDB Analysis

Lifen Cao MD PhD*, Nickolas Stabellini MS*, Christopher W. Towe MD, Xun Luo MD, Amanda L. Amin MD MS, Alberto J. Montero MD MBA

* Lifen Cao and Nickolas Stabellini contributed equally to this work.

**Correspondence:** Alberto J. Montero MD MBA

Professor of Medicine

University Hospitals Seidman Cancer Center

Case Western Reserve University School of Medicine

11100 Euclid Avenue, Lakeside Suite 1200

Cleveland OH 44106

(T) 216-844-3591

(F) 216-201-4676

(E) Alberto.Montero@UHhospitals.org
email@uni.edu

**Supplemental Table 1**. Cox proportional hazard regression for overall survival pathological stage I-III HR+HER2- breast cancer patients with 1 – 3 positive nodes, RS 20-25, and age > 50

|  | **Hazards Ratio** | **95% Conf. Int.** | | **p-value** |
| --- | --- | --- | --- | --- |
| Endochemotherapy vs. endocrine therapy alone | 0.740 | 0.556 | 0.983 | 0.038 |
| **Race** |  | | | |
| White | Reference | | | |
| African American | 0.776 | 0.475 | 1.268 | 0.312 |
| Asian or others | 0.660 | 0.271 | 1.610 | 0.361 |
| **Charlson-Deyo score** |  | | | |
| 0 | Reference | | | |
| 1 | 1.518 | 1.088 | 2.119 | 0.014 |
| 2 | 2.621 | 1.502 | 4.573 | 0.001 |
| 3 | 2.892 | 1.174 | 7.124 | 0.021 |
| **Facility Type** |  | | | |
| Community | Reference | | | |
| Comprehensive | 0.614 | 0.385 | 0.978 | 0.040 |
| Academic | 0.465 | 0.283 | 0.764 | 0.003 |
| Integrated | 0.445 | 0.263 | 0.755 | 0.003 |
| **Insurance Status** |  | | | |
| Public insurance | Reference | | | |
| Private insurance | 0.450 | 0.334 | 0.606 | <0.001 |
| Not insured | 0.957 | 0.302 | 3.032 | 0.940 |
| **Grade** |  | | | |
| Well differentiated | Reference | | | |
| Moderately differentiated | 1.186 | 0.812 | 1.732 | 0.377 |
| Poorly or undifferentiated | 1.821 | 1.177 | 2.818 | 0.007 |
| **Lympho-vascular invasion** | 1.086 | 0.817 | 1.443 | 0.570 |
| **Pathological stage** |  | | | |
| I | Reference | | | |
| II | 2.994 | 1.839 | 4.875 | <0.001 |
| III | 2.688 | 1.063 | 6.802 | 0.037 |

**Supplemental Table 2A**. Cox proportional hazard regression for overall survival pathological stage I-III HR+HER2- breast cancer patients with 1 – 3 positive nodes and RS <11

|  | **Hazards Ratio** | **95% Conf. Int.** | | **p-value** |  |  |  |
| --- | --- | --- | --- | --- | --- | --- | --- |
| **Race** |  | | | |  |  |  |
| White | Reference | | | |  |  |  |
| African American | 1.072 | 0.680 | 1.690 | 0.765 |  |  |  |
| Asian or others | 0.216 | 0.053 | 0.870 | 0.031 |  |  |  |
| **Charlson-Deyo score** |  | | | |  |  |  |
| 0 | Reference | | | |  |  |  |
| 1 | 2.006 | 1.460 | 2.755 | <0.001 |  |  |  |
| 2 | 2.674 | 1.539 | 4.644 | <0.001 |  |  |  |
| 3 | 3.875 | 1.951 | 7.699 | <0.001 |  |  |  |
| **Facility Type** |  | | | |  |  |  |
| Community | Reference | | | |  |  |  |
| Comprehensive | 0.868 | 0.511 | 1.475 | 0.601 |  |  |  |
| Academic | 0.677 | 0.387 | 1.182 | 0.170 |  |  |  |
| Integrated | 0.771 | 0.438 | 1.357 | 0.368 |  |  |  |
| **Insurance Status** |  | | | |  |  |  |
| Public insurance | Reference | | | |  |  |  |
| Private insurance | 0.353 | 0.258 | 0.484 | <0.001 |  |  |  |
| Not insured | 0.368 | 0.051 | 2.643 | 0.320 |  |  |  |
| **Grade** |  | | | |  |  |  |
| Well differentiated | Reference | | | |  |  |  |
| Moderately differentiated | 1.346 | 0.988 | 1.834 | 0.060 |  |  |  |
| Poorly or undifferentiated | 1.440 | 0.847 | 2.448 | 0.178 |  |  |  |
| **Lympho-vascular invasion** | 0.940 | 0.704 | 1.255 | 0.676 |  |  |  |
| **Pathological stage** |  | | | |  |  |  |
| I | Reference | | | |  |  |  |
| II | 2.213 | 1.492 | 3.281 | <0.001 |  |  |  |
| III | 1.408 | 0.493 | 4.027 | 0.523 |  |  |  |
| Age and treatment interactions |  | | | |  |  |  |
|  |  | | | |  |  |  |
| Age <50 & endocrine therapy alone | Reference | | | |  |  |  |
| Age <50 & endocrine plus chemotherapy | 0.620 | 0.170 | 2.258 | 0.469 |  |  |  |
| Age > 50 & endocrine therapy alone | 1.942 | 1.011 | 3.733 | 0.046 |  |  |  |
| Age>50 & endocrine plus chemotherapy | 1.212 | 0.534 | 2.753 | 0.645 |  |  |  |

**Supplemental Table 2B**. Cox proportional hazard regression for overall survival pathological stage I-III HR+HER2- breast cancer patients with 1 – 3 positive nodes and RS 12-25

|  | **Hazards Ratio** | **95% Conf. Int.** | | **p-value** |  |  |  |
| --- | --- | --- | --- | --- | --- | --- | --- |
| **Race** |  | | | |  |  |  |
| White | Reference | | | |  |  |  |
| African American | 1.226 | 0.950 | 1.581 | 0.117 |  |  |  |
| Asian or others | 0.550 | 0.317 | 0.956 | 0.034 |  |  |  |
| **Charlson-Deyo score** |  | | | |  |  |  |
| 0 | Reference | | | |  |  |  |
| 1 | 1.648 | 1.345 | 2.020 | <0.001 |  |  |  |
| 2 | 3.532 | 2.607 | 4.785 | <0.001 |  |  |  |
| 3 | 5.456 | 3.565 | 8.350 | <0.001 |  |  |  |
| **Facility Type** |  | | | |  |  |  |
| Community | Reference | | | |  |  |  |
| Comprehensive | 0.871 | 0.631 | 1.201 | 0.399 |  |  |  |
| Academic | 0.650 | 0.464 | 0.911 | 0.012 |  |  |  |
| Integrated | 0.609 | 0.428 | 0.866 | 0.006 |  |  |  |
| **Insurance Status** |  | | | |  |  |  |
| Public insurance | Reference | | | |  |  |  |
| Private insurance | 0.414 | 0.346 | 0.496 | <0.001 |  |  |  |
| Not insured | 0.896 | 0.490 | 1.639 | 0.722 |  |  |  |
| **Grade** |  | | | |  |  |  |
| Well differentiated | Reference | | | |  |  |  |
| Moderately differentiated | 1.188 | 0.970 | 1.455 | 0.095 |  |  |  |
| Poorly or undifferentiated | 1.852 | 1.437 | 2.387 | <0.001 |  |  |  |
| **Lympho-vascular invasion** | 1.141 | 0.965 | 1.349 | 0.124 |  |  |  |
| **Pathological stage** |  | | | |  |  |  |
| I | Reference | | | |  |  |  |
| II | 2.125 | 1.657 | 2.724 | <0.001 |  |  |  |
| III | 2.937 | 1.904 | 4.530 | <0.001 |  |  |  |
| Age and treatment interactions |  | | | |  |  |  |
|  |  | | | |  |  |  |
| Age <50 & endocrine therapy alone | Reference | | | |  |  |  |
| Age <50 & endocrine plus chemotherapy | 0.550 | 0.338 | 0.895 | 0.016 |  |  |  |
| Age > 50 & endocrine therapy alone | 1.214 | 0.871 | 1.692 | 0.251 |  |  |  |
| Age>50 & endocrine plus chemotherapy | 0.982 | 0.687 | 1.404 | 0.921 |  |  |  |

**Supplemental Table 3**. Cox proportional hazard regression for overall survival pathological stage I-III HR+HER2- breast cancer patients with 1 – 3 positive nodes and RS 0-25

|  | **Hazards Ratio** | **95% Conf. Int.** | | **p-value** |
| --- | --- | --- | --- | --- |
| chemotherapy vs. endocrine therapy alone | 0.647 | 0.539 | 0.777 | <0.001 |
| Age <50 vs. Age >50 | 1.461 | 1.148 | 1.861 | 0.002 |
| **Race** |  | | | |
| White | Reference | | | |
| African American | 1.164 | 0.933 | 1.454 | 0.179 |
| Asian or others | 0.462 | 0.276 | 0.771 | 0.003 |
| **Charlson-Deyo score** |  | | | |
| 0 | Reference | | | |
| 1 | 1.720 | 1.449 | 2.040 | <0.001 |
| 2 | 3.214 | 2.463 | 4.194 | <0.001 |
| 3 | 4.674 | 3.255 | 6.711 | <0.001 |
| **Facility Type** |  | | | |
| Community | Reference | | | |
| Comprehensive | 0.882 | 0.669 | 1.161 | 0.369 |
| Academic | 0.664 | 0.498 | 0.887 | 0.006 |
| Integrated | 0.667 | 0.495 | 0.900 | 0.008 |
| **Insurance Status** |  | | | |
| Public insurance | Reference | | | |
| Private insurance | 0.406 | 0.348 | 0.474 | <0.001 |
| Not insured | 0.812 | 0.456 | 1.446 | 0.480 |
| **Grade** |  | | | |
| Well differentiated | Reference | | | |
| Moderately differentiated | 1.203 | 1.015 | 1.425 | 0.033 |
| Poorly or undifferentiated | 1.675 | 1.334 | 2.104 | <0.001 |
| **Lympho-vascular invasion** | 1.093 | 0.945 | 1.264 | 0.230 |
| **Pathological stage** |  | | | |
| I | Reference | | | |
| II | 2.142 | 1.736 | 2.642 | <0.001 |
| III | 2.656 | 1.789 | 3.945 | <0.001 |
| Oncotype DX score |  | | | |
| 0-11 | Reference | | | |
| 12 | 1.122 | 0.811 | 1.551 | 0.488 |
| 13 | 1.035 | 0.748 | 1.432 | 0.834 |
| 14 | 1.351 | 1.011 | 1.806 | 0.042 |
| 15 | 0.957 | 0.681 | 1.346 | 0.802 |
| 16 | 1.153 | 0.834 | 1.595 | 0.389 |
| 17 | 1.447 | 1.073 | 1.951 | 0.015 |
| 18 | 0.916 | 0.628 | 1.336 | 0.647 |
| 19 | 1.527 | 1.098 | 2.123 | 0.012 |
| 20 | 1.700 | 1.232 | 2.346 | 0.001 |
| 21 | 2.194 | 1.631 | 2.953 | <0.001 |
| 22 | 1.966 | 1.414 | 2.733 | <0.001 |
| 23 | 1.787 | 1.204 | 2.650 | 0.004 |
| 24 | 2.028 | 1.412 | 2.912 | <0.001 |
| 25 | 2.540 | 1.751 | 3.684 | <0.001 |

**Supplemental Table 4**. Frequency distribution of patients with pathological stage I-III HR+HER2- breast cancer and 1 – 3 positive nodes

| **RS** | **Frequency** | **Percentage** | **Cumulative Percentage** |
| --- | --- | --- | --- |
| 0 to 11 | 9,259 | 32.4 | 32.4 |
| 12 | 1,731 | 6.06 | 38.46 |
| 13 | 1,891 | 6.62 | 45.07 |
| 14 | 1,862 | 6.52 | 51.59 |
| 15 | 1,833 | 6.41 | 58 |
| 16 | 1,782 | 6.24 | 64.24 |
| 17 | 1,636 | 5.72 | 69.96 |
| 18 | 1,495 | 5.23 | 75.19 |
| 19 | 1,411 | 4.94 | 80.13 |
| 20 | 1,287 | 4.5 | 84.64 |
| 21 | 1,112 | 3.89 | 88.53 |
| 22 | 1,007 | 3.52 | 92.05 |
| 23 | 887 | 3.1 | 95.15 |
| 24 | 733 | 2.56 | 97.72 |
| 25 | 652 | 2.28 | 100 |
| Total | 28,578 | 100 | |

**Supplemental Table 5**. Frequency distribution of patients with pathological stage I-III HR+HER2- breast cancer and 1 – 3 positive nodes (RS 11 to 25)

| **RS** | **Frequency** | **Percentage** | **Cumulative Percentage** |
| --- | --- | --- | --- |
| 11 | 1,596 | 7.63 | 7.63 |
| 12 | 1,731 | 8.28 | 15.91 |
| 13 | 1,891 | 9.04 | 24.95 |
| 14 | 1,862 | 8.9 | 33.85 |
| 15 | 1,833 | 8.76 | 42.62 |
| 16 | 1,782 | 8.52 | 51.14 |
| 17 | 1,636 | 7.82 | 58.96 |
| 18 | 1,495 | 7.15 | 66.11 |
| 19 | 1,411 | 6.75 | 72.85 |
| 20 | 1,287 | 6.15 | 79.01 |
| 21 | 1,112 | 5.32 | 84.32 |
| 22 | 1,007 | 4.81 | 89.14 |
| 23 | 887 | 4.24 | 93.38 |
| 24 | 733 | 3.5 | 96.88 |
| 25 | 652 | 3.12 | 100 |
